# Undifferentiated connective tissue disease: the diagnoses critically revised-experience of a single center

**DOI:** 10.1007/s10238-025-01614-1

**Published:** 2025-03-29

**Authors:** Ilaria Cavazzana, Paolo Semeraro, Cesare Tomasi, Elena Sofia Kessler, Silvia Piantoni, Alessia Caproli, Micaela Fredi, Franco Franceschini

**Affiliations:** 1 Rheumatology and Clinical Immunology Unit and Chair, Department of Experimental and Clinical Science, ERN-Reconnect Centre, ASST Spedali Civili, University of Brescia, Brescia, Italy; 2 Rheumatology and Clinical Immunology Unit and Chair, Department of Experimental and Clinical Science, University of Brescia, Brescia, Italy

**Keywords:** Undifferentiated connective tissue disease, Anti-nuclear antibodies, Systemic sclerosis, Raynaud’s phenomenon, Systemic lupus erythematosus

## Abstract

Although anti-nuclear antibodies (ANA) are considered the main entry criteria for a diagnosis of undifferentiated connective tissue disease (UCTD), many patients show different rate of ANA positivity and questionable diagnoses. Aim of the study was to revise the UCTD diagnoses and analyse the main predictors of evolution in a monocentric cohort. We retrospectively revised the diagnoses of 331 ANA positive patients, with at least one year of follow-up, classified as UCTD from 2009 and 2017. The diagnosis of UCTD was confirmed in 180 cases (54.4%). The evolution occurred in 18% of cases, after a follow-up of 6.9 (SD: 4.4) years. Raynaud’s phenomenon (RP) (OR: 2.39), puffy hands (OR: 6.3), anti-ENA (OR: 2.34), anti-Topoisomerase I antibodies (OR: 4.93), rheumatoid factor (RF) (OR: 2.86) were associated with evolution. Evolution in Systemic Lupus Erythematosus (SLE) occurred in 5 patients (2.78%) and associated with the addition of new autoantibodies, compared with other evolutions (*p*: 0.034; OR: 12; 95CI: 1.4–103.4). Evolution in Systemic Sclerosis and pSS was found in 14 (7.8%) and 8 cases (4.4%), respectively. Puffy hands and RF positivity as the predictors of SSc and pSS evolution, respectively. A confirmed diagnosis of UCTD, according with the available criteria, was assessed in about a half patients. The occurrence of puffy hands since the onset defines a patient with a potential evolution into SSc, while the addition of new specific autoantibodies represents a typical “fingerprint” of patients developing SLE.

Trial registration: Studio ANACTD np 1318.

**Supplementary Information:**

The online version contains supplementary material available at 10.1007/s10238-025-01614-1.

## Introduction

Undifferentiated connective tissue disease (UCTD) is one of the most common diagnoses made in daily rheumatology clinical practice. Old and recent papers reported that up to 20–50% of patients, presenting at a rheumatology clinic, are diagnosed as UCTD [1, 2]. However, this term frequently encounters many autoimmune or non-autoimmune clinical conditions, due to the lack of consensus on UCTD definition. To analyse the clinical features and the natural history of these patients, many efforts have been made by different authors to obtain a strict definition of the disease, modifying the anti-nuclear antibodies (ANA) titre or the duration of follow-up, as the entry criteria [1, 3–11]. Globally, UCTD is considered a defined disease, characterised by ANA and one or more signs/symptoms of well-defined connective tissue diseases (CTD), with a reasonable follow-up, to avoid misdiagnoses [12].

More recently, some authors proposed that UCTD could be considered a preclinical stage of CTD, characterised by ANA and specific inflammatory signalling or autoimmune activation pathways, leading to two main evolution profiles [13]. The progressive development of new autoantibodies and the expression of interferon-related or fibroblast activation cytokines could help to foresee the occurrence of systemic lupus erythematosus (SLE) or systemic sclerosis (SSc), respectively [13]. This approach is currently unavailable for diagnostic purpose in routine clinical practice in the majority of the third level rheumatology centres.

Starting from the formal diagnoses of UCTD made from 2009 to 2017 in our centre, our aim was to critically revise the diagnoses and identify the main predictors of evolution into CTD, namely SLE, SSc, primary Sjögren’s syndrome (pSS) and rheumatoid arthritis (RA).

## Methods

This retrospective monocentric study recruited 331 patients with a formal diagnosis of UCTD, followed-up from 2009 and 2017.

### Patients’ selection

Inclusion criteria were: the occurrence of at least one clinical feature of autoimmune systemic disease; ANA positivity, confirmed two consecutive times; at least one year of follow-up.

The exclusion criteria were: patients with follow-up of less than 1 year; occurrence of signs and symptoms of other diseases, different from autoimmune CTD; development of additional signs or autoantibodies, within 1 year of follow-up, leading to a definite diagnosis of CTD, namely SLE, SSc, RA, primary anti-phospholipid syndrome (PAPS), pSS, or mixed connective tissue disease (MCTD).

From the original 331 patients with a formal diagnosis of UCTD, 151 were excluded due to the following reasons: 86 subjects showed a shorter follow-up or a diagnosis of a non-autoimmune disease, while 65 patients showed a diagnosis of a definite CTD within 1 year of follow-up. In particular, 34 patients retrieved a diagnosis of PAPS, 16 of pSS, 7 of SSc, 4 of SLE and 4 of RA.

The final case series was composed by 180 patients with a correct (and accepted) diagnosis of UCTD. Patients were evaluated, according to our clinical practice, every 6–12 months. The laboratory tests that were used in the follow-up are listed below in methods’ section.

### Definition of CTD evolution

The evolution of UCTD into another CTD was considered at any clinical evaluation during follow-up, considering clinical and laboratory data.

In particular, the evolutions were defined with the following criteria: the 1997 ACR [14] or the 2019 ACR/EULAR criteria [15] for SLE, the 2013 ACR/EULAR criteria [16] for SSc, the 2016 [17] or the 2012 ACR/EULAR criteria [18] for pSS, the 2010 ACR/EULAR criteria [19] for RA. A diagnosis of PAPS was achieved according to two sets of criteria [20, 21], while MCTD according to the 2019 criteria [22].

### Main outcome variables

Retrospective data were collected from patients’ medical records. They included demographic data, clinical features at onset, namely fatigue, fever, arthritis, arthralgias, myalgias, photosensitivity, malar rash, subacute cutaneous lupus (SCLE), discoid lupus erythematosus (DLE) or malar rash, dermatomyositis rash, chilblain lupus, urticaria, sclerodactyly, puffy hands, mechanic’s hands, alopecia, recurrent oral ulcers; Raynaud’s phenomenon (RP), livaedo reticularis, purpuric lesions, xerostomia, xerophtalmia, parotid swelling, dyspnea, interstitial lung disease (ILD), paresthesias, migraine, dysphagia, miscarriages (at least 3 spontaneous abortions before 12nd week of gestation or 1 endouterine death at more than 12th week of gestation). Autoimmune thyroiditis was considered as accompanying feature and not as inclusion clinical criteria.

At onset the following haematological data were considered: leukopenia (white blood count < 4.000/dL), thrombocytopenia (platelet count < 150.000/dL), haemolytic anaemia with direct Coombs’ test positivity; hypergammaglobulinemia, elevation of erythrocyte sedimentation rate (ESR) and C reactive protein (CRP), complement fraction (C3 and/or C4) reduction. Autoantibodies profile at onset included ANA, detected by indirect immunofluorescence test (IFI) [23]; anti-extractable nuclear antigen (ENA) antibodies (including anti-Ro/SS-A, anti-La/SS-B, anti-U1RNP, anti-Sm, anti-CENP-B, anti-Topoisomerase I, anti-Jo1), anti-double strand (ds)DNA, anti-cyclic citrullinated peptide antibodies (ACPA); Rheumatoid factor (RF); anti-cardiolipin (aCL IgM and/or IgG), anti-β2 glycoprotein-I (anti-β2GPI IgM and/or IgG), lupus anticoagulant (LA), anti-neutrophil cytoplasm antibodies (ANCA). Autoantibody’s positivity was defined if it was confirmed two or more times, during follow-up. ANA, anti-dsDNA, anti-ENA, as well as aCL, anti-β2GPI, were performed in the same laboratory of our Centre at least at the first patients’ evaluation.

### Procedures

Every patient was clinically evaluated every six months at least for one year: during every scheduled visit the previously described variables were searched and added in the clinical chart. All patients with RP performed, during the first visit, a periungueal videocapillaroscopy (VC), according to Cutolo et al. [24]. Patients with xerophthalmia underwent an ophthalmological evaluation with the Schirmer’s test, Break-up time test (BUT) or Rose Bengal test. Patients with arthritis performed standard X-ray in order to detect erosions, according to good clinical practice.

### Ethical standards

This study has been approved by local ethics committee and it has been performed in accordance with the ethical standards laid down in the 1964 Declaration of Helsinki and its later amendments.All patients gave their informed consent prior to their inclusion in the study.

### Statistical analysis

Categorical variables were reported as proportion and/or percentage, while the continuous variables were expressed as mean (± standard deviation, SD) value. Quantitative variables were compared by Chi square test with Fisher’s correction.

The lack of Gaussian distribution of all the variables were verified by the Kolmogorov–Smirnov test.

Continuous variables were compared by Student’s t test or Wilcoxon test. Univariable analysis was performed to confirmed variable associations, multivariable analysis was performed only between groups with > 30 cases. The analysis of variance (Anova one way) was applied to compare continuous variables between different evolved groups, followed by a Bonferroni post-hoc test.

*P* values < 0.05 were considered statistically significant.

Statistical analyses were performed by Statview V.5.0.

## Results

One hundred-eighty UCTD patients, with a female to male ratio of 12:1, showed a mean age at onset of 43.1 years (SD: 15) and a mean follow-up of 8.23 years (SD: 4.9). An evolution to a defined CTD was achieved in 33 cases (18.3%) after a mean follow-up of 6.96 years (SD: 4.45). In particular, an evolution into SSc was detected in 14 patients (42.4%), into pSS in 8 patients (24.2%), into SLE in 5 patients (15.2%), into RA in 3 patients (9.1%), in PAPS and MCTD in 2 and 1 patients, respectively (6.1% and 3%).

### Clinical features at onset

The main clinical features at onset were represented by RP (46.1%), arthralgias (43.9%), fatigue (28.3%), xeropthalmia (27.2%), photosensitivity (25.5%), arthritis (23.9%), xerostomia (18.9%). Parotid swelling and confirmed hypolacrimia were found at UCTD onset in 7 patients (3.9%) and 2/49 patients (4%), respectively. Nailfold VC alterations were found in 39 patients (46.9%): most of them were non-specific (namely enlarged or tortuous loops), while 16 patients showed, since the onset, a scleroderma pattern (“early” or “active” type). Acrocyanosis, puffy hands, alopecia, myalgias, livedo reticularis, malar rash, SCLE or DLE were found in less than 9% of the global cohort.

Autoimmune thyroiditis was found in 14% of cases. Leukopenia, haemolytic anaemia and thrombocytopenia were found in 13%, 8.3% and 7%, respectively. Polyclonal hypergammaglobulinemia and reduction of complement fractions in 11% and 12.2% of cases.

### Immunological features at onset

All patients were positive for ANA, with a speckled pattern in 49.2%, homogeneous in 31.2%, nucleolar in 20.3% and centromere in 14% of cases, respectively. Isolated ANA were found only in 25 patients (13.8%), while most of patients have multiple autoantibodies’ specificities (86.1%), represented by ANA with anti-ENA or anti-dsDNA antibodies. Anti-ENA were found in 108 sera (60.5%), mainly represented by anti-Ro/SSA (66 cases; 36.7%), anti-CENP-B (18 cases; 10%), anti-U1RNP (17 cases; 9.4%).

Other disease-specific antibodies were found more rarely: anti-Sm in 3 cases (1.67%), anti-La/SSB in 12 cases (6.67%), anti-Topoisomerase I in 8 cases (4.4%) or anti-dsDNA in 15/176 cases (8.5%).

At least one test for antiphospholipid antibodies was positive in 44% of sera, represented by anti-beta2GPI (36%), aCL (24%) and LA (21%). In addition, RF and ACPA were found in 26.7% and 21% of sera.

### Comparison between evolved and stable UCTD

Evolution in a defined CTD was achieved in 33 patients (18.3%): SSc in 14 (42%), pSS in 8 (24%), and SLE in 5 cases (15%). Demographic features of the two groups (CTD evolved or not) are described in Supplemental data (supplemental Table S1): most of the patients were female and Caucasians, with a comparable mean age at onset between groups (41.4 and 43.5 years in evolved and stable UCTD, respectively) and a comparable follow-up duration (6.9 and 8.5 years, respectively).

As shown in Table 1, at the disease onset, evolved patients more frequently showed RP (*p*: 0.033; OR: 2.39) and puffy hands (*p*:0.006; OR: 6.31). No other clinical differences were found between groups. VC was performed only in patients with Raynaud’s phenomenon (21 in evolved and 62 in stable cases): VC alterations were equally found in both groups, also when considering non-specific changes and scleroderma pattern features.

**Table 1:** Disease-onset clinical features of evolved and stable UCTD patients

	Evolved n.33 (%)	Stable n.147 (%)	p	OR (95CI)
F:M	31:2 (93.9)	135:12 (91.8)	1	
Mean age at onset, years (SD)	41.45 (13.4)	43.54 (15.5)	0.47	
RP	21 (63.64)	62 (42.18)	0.033	**2.39 (1.09–5.24)**
VC SSc-specific pattern	11/21 (52.4)	25/62 (40.32)	0.44	
Xerophtalmia	12 (36.36)	37 (25.17)	0.200	
Arthralgias	11 (33.33)	68 (46.26)	0.244	
Xerostomia	8 (24.24)	26 (17,68)	0.460	
Photosensitivity	7 (21.21)	39 (26.53)	0.660	
Arthritis	6 (18.18)	37 (25.17)	0.501	
Puffy hands	6 (18.18)	5 (3.40)	0.006	**6.31 (1.8–22.1)**
Acrocyanosis	5 (15.15)	12 (8.16)	0.205	
Autoimmune thyroiditis	4 (12.12)	22 (14.97)	0.790	
Alopecia	3 (9.09)	7 (4.76)	0.394	
Discoid lupus erythematosus	2 (6.06)	6 (4.08)	0.640	
Livedo reticularis	3 (9.10)	6 (4.08)	0.369	
Parotid swelling	2 (6.06)	5 (3.40)	0.614	

*RP* Raynaud’s phenomenon; *VC* videocapillaroscopy. *Ssc* systemic sclerosis. In bold statistically significant values with their 95% confidence interval

Evolved patients showed more frequent hypergammaglobulinemia (21% vs 9.5%; *p*: 0.07) compared with stable UCTD patients, while no differences in complement reduction, leukopenia, anaemia or thrombocytopenia were found between groups (Supplemental Table S2).

The autoantibody profile was reported in Table 2: anti-ENA positivity was more frequent in evolved cases, in particular, the positivity for anti-Topoisomerase I antibodies (*p*: 0.039; OR: 4.93) and for RF (*p*: 0.051; OR: 2.86). No differences in anti-dsDNA, aCL or anti-beta2 GPI positivity was found between groups. More than 90% of evolved UCTD showed multiple ANA specificities, without significant differences compared with stable UCTD. Multiple ANA were represented by ANA and single anti-ENA in 23 sera (76.7%), ANA and multiple anti-ENAs in 2 sera (6.7%) and ANA and anti-dsDNA in 3 sera (10%).

**Table 2:** Autoantibodies’ profile of evolved and stable UCTD patients

	Evolved n.33 (%)	Stable n. 147 (%)	p	OR (95CI)
ANA	33 (100)	147 (100)	1	
Isolated ANA	3 (9.09)	30 (20.41)	0.211	
Multiple ANA	30 (90.91)	117 (79.59)	0.211	
Anti-ENA total	25 (75.76)	84 (57.14)	0.051	2.34 (0.99–5.5)
Anti-Ro/SS-A	10 (30.30)	56 (38.10)	0.432	
Anti-CENP-B	6 (18.18)	12 (8.16)	0.106	
Anti-Topoisomerase I	4 (12.12)	4 (2.72)	0.039	**4.93 (1.17–20.8)**
Anti-U1RNP	4 (12.12)	13 (8.84)	0.521	
Anti-La/SS-B	1 (3.03)	11 (7.48)	0.698	
Anti-Sm	1 (3.03)	2 (1.36)	0.457	
aCL (IgG and/or IgM)	6/28 (21.43)	29/117 (24.79)	0,810	
aβ2GPI (IgG and/or IgM)	11/28 (39.29)	40/112 (35.71)	0.437	
LA	2/19 (10.53)	19/81 (2.46)	0.348	
Anti-dsDNA	3/32 (9.38)	12/144 (8.33)	1	
ACPA	4/11 (36.36)	7/41 (17.07)	0.216	
RF	9/20 (45)	18/81 (22.22)	0.051	**2.86 (1.03–7.98)**
ANCA	0/33 (0)	2/145 (1.38)	1	

*ANA* antinuclear antibodies; *anti-ENA* anti-extractable nuclear antigen antibodies; *aCL* anti-cardiolipin antibodies; *anti-β2GPI* anti-β2 glycoprotein-I antibodies; *LA* lupus anticoagulant; *Anti-dsDNA* anti-double strand DNA antibodies; *ACPA* anti-cyclic citrullinated peptide antibodies; *RF* rheumatoid factor; *ANCA* anti-neutrophil cytoplasm antibodies. In bold statistically significant values with their 95% confidence interval

Univariate analysis confirms the significant associations between evolution and RP (*p*:0.025), puffy hands (*p*:0.001), anti-ENA positivity (*p*:0.048), anti-Topoisomerase I (*p*:0.018) and RF (*p*:0.040). On the other hand, multivariable analysis, including age at onset and sex distribution, confirms RF as the only item associated with evolution (*p*:0.035; OR: 3.6; 95CI: 1.1–12.2).

#### Systemic lupus erythematosus

Evolution in SLE is a quite rare event, occurred in 5 patients (2.78%) after a mean of 7.2 years (SD: 3) of follow-up. Comparing 5 UCTD-SLE patients and other 147 stable UCTD, we found that a younger age at onset (p: 0.05), alopecia (p: 0.029; OR: 13.3), nucleolar ANA pattern (p: 0.029; OR: 12.8) and anti-dsDNA (p: 0.007; OR: 16.5) positivity were features associated with evolution, as shown in Table 3.

**Table 3:** Demographic, clinical and serological data associated with SLE evolution

	Evolved in SLE n. 5 (%)	Stable UCTD n. 147 (%)	p	OR (95CI)
Mean age at onset, years (SD)	30 (5.1)	43.54 (15.5)	0.05	
Alopecia	2 (40)	7 (4.76)	0.029	**13.3 (1.91–93)**
ANA nucleolar pattern	3 (75)	19 (19)	0.029	**12.8 (1.26–129.8)**
Anti-dsDNA	3 (60)	12/144 (8.33)	0.007	**16.5 (2.5–108.6)**

*ANA* antinuclear antibodies; *Anti-dsDNA* anti-double strand DNA antibodies. In bold statistically significant values with their 95% confidence interval, when appropriated

The evolution was characterised by the new onset of arthritis (n.3), glomerulonephritis (n.2) and pleurisy (n.1), anti-dsDNA (n.2), positive direct Coombs test (1 case), complement reduction (1 case), lymphopenia (n.1). Univariate analysis confirms the significant association between SLE evolution and lower age at onset (p:0.05), alopecia (p:0.001), anti-dsDNA (p:0.002), and nucleolar ANA pattern (p:0.001). The development of new autoantibody specificity was observed in 3/5 evolved in SLE (60%), 1/14 evolved in SSc (7%) and 1/8 evolved in pSS (12.5%).The addition of autoantibodies is significantly associated only with SLE evolution compared with other evolutions (p: 0.034; OR: 12; 95CI: 1.4–103.4).

#### Systemic Sclerosis

Evolution in SSc was found in 14 cases (7.78%), after a mean follow-up of 5.62 years (SD:5). No demographic data were associated with SSc evolution. Clinical and serological associations were summarised in Table 4: RP, VC alterations, puffy hands and acrocyanosis at onset were significantly associated with SSc evolution.

**Table 4:** Clinical and serological data associated with progression to SSc

	Evolved in SSc n. 14 (%)	Stable UCTD n. 147 (%)	p	OR (95CI)
RP	14 (100)	62 (42.18)	0.00001	**Infinite**
VC SSc-specific pattern	11/14 (78.57)	25/147 (17.01)	< 0.00001	**17.9 (4.6–68.8)**
Puffy hands	6 (42.86)	5 (3.40)	0.00005	**21.3 (5.3–16.5)**
Acrocyanosis	4 (28.57)	12 (8.16)	0.036	**4.5 (1.2–16.5)**
Anti-ENA total	12 (85.71)	84 (57.14)	0.046	4.5 (0.9–20.8)
Anti-CENP-B	5 (35.71)	12 (8.16)	0.008	**6.25 (1.8–21.6)**
Anti-Topoisomerase I	4 (28.57)	4 (2.72)	0.002	**14.3 (3.1–65.8)**
Anti-Ro/SS-A	1 (7.14)	56 (38.10)	0.020	**0.13 (0.02–0.98)**

RP: Raynaud’s phenomenon; VC: videocapillaroscopy; Ssc: Systemic sclerosis; anti-ENA: anti-extractable nuclear antigen antibodies. In bold statistically significant values with their 95% confidence interval

Analysis of VC changes showed that both SSc pattern and non-specific changes were significantly associated with SSc evolution: SSc pattern was present in 5/9 (55.5%) SSc-evolved and 10/147 (6.8%) stable UCTD patients (*p*: 0.004; OR: 7.6; 95CI: 2.1–27); non-specific alterations were found in 6/14 (42.8%) of SSc-evolved and 15/147 (10.2%) of stable UCTD (p: 0.004; OR: 6.6; 95CI: 2–21.6).

In addition, SSc-specific autoantibodies, such as anti-CENP-B and anti-Topoisomerase I were predictive of evolution. In contrast, the occurrence of anti-Ro/SSA is a protective factor for progression to SSc.

Univariate analysis confirms the association between SSc evolution and RP (*p* < 0.0001), VC changes (*p* < 0.0001), puffy hands (*p* < 0.0001), acrocyanosis (*p*:0.015), anti-ENA positivity (*p*:0.038), anti-CENP-B (*p*:0.001), anti-Topoisomerase-I (*p* < 0.0001). A negative association was confirmed with anti-Ro/SSA (*p*:0.021). Multivariable analysis was not performed due to the low number of evolved cases. Puffy hands showed the higher OR for evolution.

We perform a sub-analysis of patents with RP from the global cohort: above 83 patients with RP, 12 could be classified as very early SSc (14.4%), according to VEDOSS criteria [25]. They are characterized by RP, no specific VC alterations and no SSc-specific autoantibodies. Evolution into CTD occurred in only 2 patients (16.7%): 1 in SSc and 1 in pSS. Other 10 patients remained stable UCTD, after 8.39 years of follow-up (SD: 2.34).

#### Primary Sjogren’s syndrome

An evolution into pSS was found in 8 cases (4.4%), after a mean follow-up of 8.8 years (SD: 5.4). Patients evolved in pSS more frequently showed at onset xerostomia (*p*: 0.009; OR: 7.8), xeropthalmia (*p*: 0.034; OR: 4.96), parotid swelling (*p*: 0.043; OR: 9.5), hypergammaglobulinemia (*p*: 0.044; OR: 5.7) and RF positivity (*p*: 0.001; OR: 21). Anti-Ro/SSA and anti-La/SSB antibodies were more frequent in UCTD evolved in pSS, but without significant difference compared with stable UCTD (Table 5).

**Table 5:** Clinical and serological data associated with progression to pSS

	Evolved in pSS n. 8 (%)	Stable UCTD n. 147 (%)	p	OR (95CI)
Xerostomia	5 (62.5)	26 (17.69)	0.009	**7.8 (1.7–35.4)**
Xerophtalmia	5 (62.5)	37 (25.17)	0.034	**4.96 (1.1–21.75)**
Parotid swelling	2 (25)	5 (3.40)	0.043	**9.5 (1.53–59.5)**
Hypergammaglobulinemia	3 (37.50)	14 (9.52)	0.044	**5.7 (1.23–26.4)**
Anti-Ro/SS-A	6 (75)	56 (38.10)	0.060	
Anti-La/SS-B	1 (12.50)	11 (7.48)	0.483	
RF	6/7 (85.71)	18/81 (22.22)	0.001	**21 (2.37–185.9)**
Multiple autoantibodies	8 (100)	116 (78.9)	0.36	
Anti-ENA total	7 (87.5)	81 (55)	0.14	

*RF* rheumatoid factor; *anti-ENA* anti-extractable nuclear antigen antibodies. In bold statistically significant values with their 95% confidence interval

All patients evolved in pSS showed since the onset multiple autoantibodies’ specificities: 87.5% of them are anti-ENA positive and 75% anti-Ro positive. At the evolution, 2 additional patients developed anti-Ro/SSA, that resulted to be positive in all 8 pSS patients. Another patient developed anti-La/SSB, RF and hypergammaglobulinemia. Univariate analysis confirms a significant association between pSS evolution and xerostomia (*p*:0.002), xeropthalmia (*p*:0.02), parotid swelling (*p*:0.004), hypergammaglobulinemia (*p*:0.013) and RF (*p* < 0.001). RF showed the higher OR for evolution in pSS.

#### Rheumatoid arthritis

An evolution in RA occurred in 3 patients (1.67%). No demographic or clinical features resulted to be associated to evolution. Only RF and ACPA antibodies represent the serological items predictive for evolution (*p*: 0.019 and 0.009, respectively) (Supplemental Table S3A and B).

#### Comparison between different CTDs

No differences in clinical features were found between three different groups of evolved patients (SLE, pSS and SSc). All three groups showed a comparable age at onset, by Anova analysis with Bonferroni correction. Moreover, the timing of evolutions into different CTDs was comparable between different group. Nevertheless, SSc patients evolved earlier compared with other evolved cases (supplemental Table S4): at 5 years of follow-up since UCTD diagnoses, evolution in SSc occurred in 65% of patients, while evolution in pSS and SLE occurred only in 37.5% and 20% of cases, respectively.

As expected, SSc evolved patients more frequently onset with RP, but no other differences were found with the other evolutions. Patients with SSc showed more rarely hypergammaglobulinemia and anti-Ro/SSA, while the same rate of multiple autoantibodies, aPL or anti-ENA positivities was found in all three groups of evolved cases (supplemental Table S5).

During follow-up, only one patient died and 4 patients were in stable remission.

## Discussion

The present study reviewed the diagnosis of UCTD of 311 consecutive patients and analysed the different clinical and immunological “footprints” of patients with a confirmed diagnosis.

Although ANA positivity represented a clear entry-criteria in all original 331 UCTD cases, the diagnosis was confirmed only in 180 cases (54.4%). The other patients resulted to have different disorders (such as, infectious diseases, or post-infective clinical features) or developed a full-blown CTD within one year of follow-up. The high rate of misdiagnosis reflects the high range of evolution, remission and stability rates of UCTD cohorts, recently reported [26]. This is due to variable rate of ANA positivity, different follow-up duration or variable clinical pictures as the inclusion criteria for UCTD diagnosis considered in old and recent published papers. Some authors considered as “UCTD” cases patients without ANA positivity [8] or with clinical features suggestive for SLE [27, 28] or early SSc [3, 25] since the onset, obtaining a wide range of evolution rates. These differences account also for the low grade of ratings for many predictors of evolution either from the clinical and the immunological sides [26].

In this paper we applied strict inclusion criteria, according with the original Mosca’s preliminary criteria [6] with ANA positivity in 100% of cases, and a minimum follow-up of one year [9, 11]. Patients with a diagnosis of well-defined CTD or who showed the appearance of additional signs or new disease-specific autoantibodies, within one year of onset, were excluded. Although no definite exclusion criteria have been published, in clinical practice the addition of new ANA specificities [29] or the occurrence of some specific clinical features of CTDs [30] are considered parts of an evolving picture towards a well-defined CTD. Moreover, some authors considered some disease specific autoantibodies, such as anti-dsDNA, anti-Sm, anti-P-proteins or SSc-specific ANA, as highly predictive for evolution [5, 30, 31].

The rate of evolution observed in our cases was of 18%, in line with other cohorts [from 12], after a follow-up of 6.9 (SD: 4.4) years. The main symptoms at UCTD onset are perfectly in line with others, reviewed by Rubio et al. [12], represented by RP, arthralgias, sicca, and photosensitivity. RP, reported in 63% of evolved patients, is predictive of evolution in CTD, as well as puffy hands in univariate analysis. However, specific and non-specific VC changes are not associated with evolution to CTD.

Despite the high frequency of RP, as in most published UCTD cohorts, the evolution to SSc occurs only in 7.8% of our UCTD patients, in line with what is already known [31], but represents approximately half of all patients progressing to a defined CTD. The SSc evolution is associated to multiple items by univariate analysis: puffy hands predict SSc evolution with higher probability.

RP is globally considered a marker of SSc evolution [8, 9, 25, 32, 33], as well as puffy hands [33]. Recently, rather than the single items, different authors proposed that the combinations of puffy hands, SSc-specific VC changes and SSc-specific autoantibodies could be considered highly predictive for SSc evolution [25, 26, 33, 34]. VEDOSS criteria, in fact, identify patients with a very early diagnosis of SSc, characterized by RP combined with puffy fingers, VC alterations and SSc-specific autoantibodies [25, 34]. The accrual of these items gives to the patients a high risk to progress into SSc within 5 years [34]. Similar conclusions were previously reported by Koenig et al. [33], that defined a higher risk to develop a SSc in patients with specific VC alterations and SSc specific antibodies. By contrast patients with “pre-CTD”, namely with RP, non-specific ANA and non-specific VC alterations, evolved into SSc only in 3.4% of cases.

Basing on these data, we performed a sub-analysis of our 83 patents with RP. Twelve patients have been re-classified as very early SSc (14.4%), according to VEDOSS criteria [25]. These cases are characterized by RP, non-specific VC alterations and non SSc-specific autoantibodies. Evolution into CTD occurred in only 2 patients (16.7%): 1 in SSc and 1 in pSS. Other 10 patients remained stable UCTD, after 8.39 years of follow-up (SD: 2.34). In our hands UCTD patients with RP should be considered “at risk to develop” SSc, but with a low rate of SSc evolution (8%), according to other Authors [33].

In the present cohort anti-Ro/SSA antibodies are very common and equally distributed between stable and evolved UCTD. Anyway, it resulted a protective factor for SSc evolution by univariate analysis. This autoantibody is reported to be associated to evolution in SLE with a very low grade of evidence [26] or to evolution in pSS [12], but no data regarding its role in SSc evolution are published. Within SSc, many Authors reported a possible association between anti-Ro 52 and severe pulmonary involvement [35–37]. In our sera, no data were disposable regarding the anti-Ro 52 reactivity.

Evolution in SLE occurred in 2.7% of UCTD and in 15% of evolved patients. This low rate is probably due to the strict exclusion criteria applied in the definition of UCTD cohort and to the rare occurrence of SLE specific autoantibodies, such as anti-dsDNA and anti-Sm both in SLE-evolved and in stable cases. Some authors define UCTD as an autoimmune condition with ANA, non-specific autoantibodies (i.e. anti-Ro/SSA) and non-specific clinical features (arthralgia and RP), while, in the “autoimmune” route to SLE, UCTD patients progressively developed malar rash and new SLE-specific autoantibodies, such as anti-Sm and anti-dsDNA [27, 29]. Accordingly, our 5 UCTD evolved in SLE developed specific autoantibodies during follow-up in 75% of cases: anti-dsDNA (n.2), anti-Sm (n.1) and anti-ribosomal antibodies (n.1). Anti-dsDNA represented the autoantibody found in all SLE-evolved UCTD cases. The addition of specific autoantibodies peculiarly characterizes the autoimmune spreading phenomenon, occurring during the evolution of UCTD patients into SLE [29]. In our cohort, the addition of autoantibodies, as expression of epitope spreading, is significantly associated only with SLE evolution compared with other evolutions (p: 0.034; OR: 12; 95CI: 1.4–103.4).

Evolution in pSS occurred in 4.4% of all UCTD and in 24% of evolved cases. As expected, xerostomia, xerophtalmia, parotid swelling, hypergammaglobulinemia and RF represented predictors for evolution by univariate analysis. Anti-Ro/SSA antibodies are positive in all UCTD evolved in pSS at the moment of evolution, confirming that it represents the autoimmune marker of this disease. This data is in line with other authors reported a prevalent evolution in pSS of UCTD patients with anti-Ro/SSA positivity [4, 7, 8, 11, 31].

Few patients evolved into RA, as expected from entry criteria, confirming the low rate of RA evolution also in other cohorts [12]: RF and ACPA represent two markers associated to evolution. Surprisingly, arthritis does not represent a predictor of progression, as it occurs in approximately 20% of both evolved and stable UCTD.

RF positivity represents in our cohort the only serological marker of evolution in CTD, as well as in pSS. This result confirms the association, although very low, between RF and CTD evolution reported in the review by Dyball et al. [26]. The association between pSS evolution and RF is not surprising, given the high rate of RF in pSS as well as hypergammaglobulinemia.

Our work showed some limitations, represented by the small number of patients enrolled; the retrospective and monocentric design of the study. Nevertheless, the diagnoses of UCTD have been accurately reviewed, and the autoantibody profile has been performed in the same laboratory. The monocentric detection of ANA and anti-dsDNA is a critical point for many reasons. The definition of “ANA positivity”, primary entry criteria for UCTD, directly depends on the starting dilution of the serum, as well as on the subjective experience of the operator. The performance of ANA test in the same laboratory ensures a correct detection, according to international guidelines [23, 38]. Moreover, anti-dsDNA testing is considered a crucial point, mainly because not all the commercial assays available are considered appropriate in terms of sensitivity, specificity and positive predictive value [38, 39].

In conclusion, a correct diagnosis has been assessed in about a half of patients, previously classified as UCTD. An evolution occurred in 18% of cases after a long follow-up, while 43% of excluded cases developed en evolution in CTD before 1 year after the clinical onset. UCTD could be considered a preliminary diagnosis, that should be reevaluated at every visit.

Due to the low number of cases, a multivariate analysis wasn’t performed for single groups of evolutions. Anyway, the occurrence of puffy hands since the onset defines a patient with a potential evolution into SSc, while the addition of new specific autoantibodies represents a typical “fingerprint” of patients developing SLE.

In order to accurately define the potential evolution of a UCTD patient, the possibility of dosing different biomarkers of immune activation could help to identify the patients at risk, before the clinical onset of specific symptoms [13, 40–42]. The combination of cytokines’ profile and autoantibodies could represent the optimal tool to correctly define different types of UCTDs, managing their specific follow-up during time.

## Supplementary Information

Below is the link to the electronic supplementary material.

Supplementary file1 (DOCX 24 KB)

## Data Availability

No datasets were generated or analysed during the current study.

## References

[1] Alarcon GS, Williams GV, Singer JZ, Steen VD, Clegg DO, Paulus HE, et al. Early undifferentiated connective tissue disease.I. Early clinical manifestation in a large cohort of patients with undifferentiated connective tissue diseases compared with cohorts of well established connective tissue disease. J Rheumatol. 1991;18:1332–9. 1757934

[2] Antunes M, Scire CA, Talarico R, Alexander T, Avcin T, Belocchi C, Doria A, et al. Undifferentiated connective tissue disease: state of the art on clinical practice guidelines. RMD Open. 2018;4:e000786. 10.1136/rmdopen-2018-000786. 30886731 10.1136/rmdopen-2018-000786 PMC6397427

[3] Leroy EC, Maricq HR, Bashar Kahaleh M. Undifferentiated connective tissue syndromes. Arthritis Rheum. 1980;23:341–3. 7362686 10.1002/art.1780230312

[4] Mosca M, Tavoni A, Neri R, Bencivelli W, Bombardieri S. Undifferentiated connective tissue diseases: the clinical and serological profiles of 91 patients followed for at least 1 year. Lupus. 1998;7:95–100. 9541093 10.1191/096120398678919787

[5] Danieli MG, Fraticelli P, Salvi A, Gabrielli A, Danieli G. Undifferentiated connective tissue disease: natural history and evolution into definite CTD assessed in 84 patients initially diagnosed as early UCTD. Clin Rheumatol. 1998;17:195–201. 9694051 10.1007/BF01451046

[6] Mosca M, Neri R, Bombardieri S. Undifferentiated connective tissue diseases (UCTD): a review of the literature and a proposal for preliminary classification criteria. Clin Exp Rheumatol. 1999;17:615–20. 10544849

[7] Cavazzana I, Franceschini F, Belfiore N, Quinzanini M, Caporali R, Calzavara-Pinton PG, Bettoni L, Brucato A, Cattaneo R, Montecucco CM. Undifferentiated connective tissue disease with antibodies to Ro/SSa: clinical features and follow-up of 148 patients. Clin Exp Rheumatol. 2001;19:403–9. 11491495

[8] Bodolay E, Csiki Z, Szekanecz Z, Ben T, Kiss E, Zeher M, Szuchs G, Dankò K, Szegedi G. Five-year follow-up of 665 Hungarian patients with undifferentiated connective tissue disease (UCTD). Clin Exp Rheumatol. 2003;21:313–20. 12846049

[9] Guerrero LF, Rueda JC, Arciniegas R, Rueda JM. Undifferentiated connective tissue disease in a rheumatology centre in Cali, Colombia: clinical features of 94 patients followed for a year. Rheumatol Int. 2013;33:1085–8. 10.1007/s00296-011-2234-y. 22116525 10.1007/s00296-011-2234-y

[10] Garcia-Gonzalez M, Rodriguez-Lozano B, Bustabad S, Ferraz-Amaro I. Undifferentiated connective tissue disease: predictors of evolution into definite disease. Clin Exp Rheumatol. 2017;35:739–45. 28770704

[11] Radin M, Rubini E, Cecchi I, Foddai SG, Barinotti A, Rossi D, Sciascia S, Roccatello D. Disease evolution in a long-term follow-up of 104 undifferentiated connective tissue disease patients. Clin Exp Rheumatol. 2022;40:575–80. 10.55563/clinexprheumatol/7vp1bo. 34251309 10.55563/clinexprheumatol/7vp1bo

[12] Rubio J, Kyttaris VC. Undifferentiated connective tissue disease: comprehensive review. Curr Rheumatol Rep. 2023;25:98–106. 10.1007/s11926-023-01099-5. 36884206 10.1007/s11926-023-01099-5

[13] Pope JE, Calderon LM. Precursors to systemic sclerosis and systemic lupus erythematosus: from undifferentiated connective disease to the development of identifiable connective tissue diseases. Front Immunology. 2022;13:869172. 10.3389/fimmu.2022.869172. 10.3389/fimmu.2022.869172 PMC9118990 35603174

[14] Hochberg MC. Updating the American College of rheumatology revised criteria for the classification of systemic lupus erythematosus. Arthritis Rheum. 1997;40:1725. 10.1002/art.1780400928. 9324032 10.1002/art.1780400928

[15] Aringer M, Costenbader K, Daikh D, et al. 2019 European league against rheumatism/American college of rheumatology classification criteria for systemic lupus erythematosus. Arthritis Rheumatol. 2019;71:1400–12. 10.1002/art.40930. 31385462 10.1002/art.40930 PMC6827566

[16] van den Hoogen F, Khanna D, Fransen J, et al. 2013 classification criteria for systemic sclerosis: an American college of rheumatology/European league against rheumatism collaborative initiative. Ann Rheum Dis. 2013;72:1747–55. 24092682 10.1136/annrheumdis-2013-204424

[17] Shiboski CH, Shiboski SC, Seror R, et al. 2016 American College of rheumatology/European league against rheumatism classification criteria for primary sjögren’s syndrome: a consensus and data-driven methodology involving three international patient cohorts. Arthritis Rheumatol. 2017;69:35–45. 27785888 10.1002/art.39859 PMC5650478

[18] Shiboski SC, Shiboski CH, Criswell LA, et al. American College of rheumatology classification criteria for Sjögren’s syndrome: a data-driven, expert consensus approach in the Sjögren’s international collaborative clinical alliance cohort. Arthritis Care Res (Hoboken). 2012;64:475–87. 22563590 10.1002/acr.21591 PMC3349440

[19] Aletaha D, Neogi T, Silman AJ, et al. 2010 Rheumatoid arthritis classification criteria: an American College of Rheumatology/European League against rheumatism collaborative initiative. Arthritis Rheum. 2010;62:2569–81. 10.1002/art.27584. 20872595 10.1002/art.27584

[20] Miyakis S, Lockshin MD, Atsumi T, et al. International consensus statement on an update of the classification criteria for definite antiphospholipid syndrome (APS). J Thromb Haemost. 2006;4:295–306. 10.1111/j.1538-7836.2006.01753.x. 16420554 10.1111/j.1538-7836.2006.01753.x

[21] Barbhaiya M, Zuily S, Naden R, et al. 2023 ACR/EULAR antiphospholipid syndrome classification criteria. Arthritis Rheumatol. 2023;75:1687–702. 10.1002/art.42624. 37635643 10.1002/art.42624

[22] Tanaka Y, Kuwana M, Fujii T, et al. 2019 Diagnostic criteria for mixed connective tissue disease (MCTD): from the Japan research committee of the ministry of health, labor, and welfare for systemic autoimmune diseases. Mod Rheumatol. 2021;31:29–33. 10.1080/14397595.2019.1709944. 31903831 10.1080/14397595.2019.1709944

[23] Meroni PL, Schur PH. ANA screening: an old test with new recommendations. Ann Rheum Dis. 2010;69:1420–2. 10.1136/ard.2009.127100. 20511607 10.1136/ard.2009.127100

[24] Cutolo M, Pizzorni C, Secchi ME, Sulli A. Capillaroscopy. Best Pract Res Clin Rheumatol. 2008;22:1093–108. 10.1016/j.berh.2008.09.001. 19041079 10.1016/j.berh.2008.09.001

[25] Minier T, Guiducci S, Bellando-Randone S, Bruni C, Lepri G, Czirjak L, et al. Preliminary analysis of the very early diagnosis of systemic sclerosis (VEDOSS) EUSTAR multicentre study: evidence for puffy fingers as a pivotal sign for suspicion of systemic sclerosis. Ann Rheum Dis. 2014;73:2087–93. 10.1136/annrheumdis-2013-203716. 23940211 10.1136/annrheumdis-2013-203716

[26] Dyball S, Rodziewicz M, Mendoza-Pinto C, Bruce IN, Parker B. Predicting progression from undifferentiated connective tissue disease to definite connective tissue disease: a systematic review and meta-analysis. Autoimmun Rev. 2022;21:103184. 10.1016/j.autrev.2022.103184. 36031048 10.1016/j.autrev.2022.103184

[27] Sciascia S, Roccatello D, Radin M, Parodis I, Yazdany J, Pons-Estel G, Mosca M. Differentiating between UCTD and early-stage SLE: from definitions to clinical approach. Nat Rev Rheumatol. 2022;18:9–21. 10.1038/s41584-021-00710-2. 34764455 10.1038/s41584-021-00710-2

[28] Swaak AJK, van de Brink H, Smeenk RJT, Manger K, Kalden JR, Tosi S, et al. Incomplete lupus erythematosus: results of a multicentre study under the supervision of the EULAR standing committee on international clinical studies including therapeutic trials (ESCISIT). Rheumatology. 2001;40:89–94. 10.1093/rheumatology/40.1.89. 11157147 10.1093/rheumatology/40.1.89

[29] Arbuckle MR, McClain MT, Rubertone MV, et al. Development of autoantibodies before the clinical onset of systemic lupus erythematosus. N Engl J Med. 2003;349:1526–33. 10.1056/NEJMoa021933. 14561795 10.1056/NEJMoa021933

[30] Doria A, Mosca M, Gambari PF, Bombardieri S. Defining unclassifiable connective tissue diseases: incomplete, undifferentiated, or both? J Rheumatol. 2005;32:213–5. 15693073

[31] Calvo-Alen J, Alarcon GS, Burgard SL, Burst N, Bartolucci AA, Williams HJ. Systemic lupus erythematosus: predictors of its occurrence among a cohort of patients with early undifferentiated connective disease: multivariate analyses and identification of risk factors. J Rheumatol. 1996;23:469–75. 8832985

[32] Valentini G, Pope JE. Undifferentiated connective tissue disease at risk for systemic sclerosis: Which patients might be labeled prescleroderma? Autoimmun Rev. 2020;19:102659. 10.1016/j.autrev.2020.102659. 32942034 10.1016/j.autrev.2020.102659

[33] Koenig M, Joyal F, Fritzler MJ, Roussin A, Abrahamowicz M, Boire G, et al. Autoantibodies and microvascular damage are independent predictive factors for the progression of Raynaud’s phenomenon to systemic sclerosis: a twenty-year prospective study of 586 patients, with validation of proposed criteria for early systemic sclerosis. Arthritis Rheum. 2008;58:3902–12. 10.1002/art.24038. 19035499 10.1002/art.24038

[34] Bellando Randone S, Del Galdo F, Lepri G, Minier T, Huscher D, Furst D, et al. Progression of patients with Raynaud’s phenomenon to systemic sclerosis classified according to the 2013 ACR/EULAR criteria: five year analysis of the EUSTAR multicentre prospective study for very early diagnosis of systemic sclerosis (VEDOSS). Lancet Rheumatol. 2021;3:e834–43. 38287630 10.1016/S2665-9913(21)00244-7

[35] Hudson M, Pope J, Mahler M, Tatibouet S, Steel R, Baron M, Fritzler MJ. Clinical significance of antibodies to Ro52/TRIM21 in systemic sclerosis. Arthr Res Ther. 2012;14:R50. 10.1186/ar3763. 22394602 10.1186/ar3763 PMC3446416

[36] Wodkowski M, Hudson M, Proudman S, Walker J, Stevens W, Nikpiur M, et al. Monospecific anti-Ro52/TRIM21 antibodies in a tri-nation cohort of 1574 systemic sclerosis subjects: evidence of an association with interstitial lung disease and worse survival. Clin Exp Rheum. 2015;33:S131–5. 26315678

[37] Lee A, Patterson KA, Tan DJ, Wilson ME, Proudman SM, Stevens W, et al. Anti-Ro52/TRIM21 is independently associated with pulmonary arterial hypertension and mortality in a cohort of systemic sclerosis patients. Scand J Rheumatol. 2021;50:469–74. 10.1080/03009742.2021.1887927. 33851896 10.1080/03009742.2021.1887927

[38] Agmon-Levin N, Damoiseaux J, Kallenberg C, Sack U, Witte T, Herold M, et al. International recommendations for the assessment of autoantibodies to cellular antigens referred to as anti-nuclear antibodies. Ann Rheum Dis. 2014;73:17–23. 10.1136/annrheumdis-2013-203863. 24126457 10.1136/annrheumdis-2013-203863

[39] Isenberg DA, Dudeney C, Williams W, Addison I, Charles S, Clarke J, Todd-Pokropek A. Measurement of anti-DNA antibodies: a reappraisal using five different methods. Ann Rheum Dis. 1987;46:448–56. 10.1136/ard.46.6.448. 3498446 10.1136/ard.46.6.448 PMC1002163

[40] van Bon L, Affandi AJ, Broen J, Christmann RB, Marijnissen RJ, Stawski L, Farina GA, et al. Proteome-wide analysis and CXCL4 as a biomarker in systemic sclerosis. N Engl J Med. 2014;370:433–43. 10.1056/NEJMoa1114576. 24350901 10.1056/NEJMoa1114576 PMC4040466

[41] Lande R, Mennella A, Palazzo R, et al. Anti-CXCL4 antibody reactivity is present in systemic sclerosis (SSc) and correlates with the SSc type I interferon signature. Int J Mol Sci. 2020;21:5102. 10.3390/ijms21145102. 32707718 10.3390/ijms21145102 PMC7404208

[42] Mennella R, Palazzo A, Pietraforte I, Stefanantoni K, Iannace N, Butera A, et al. Anti-CXCL4 antibody reactivity is present in systemic sclerosis (SSc) and correlates with the SSc type I interferon signature. Int J Mol Sci. 2020;21:5102. 10.3390/ijms21145102. 32707718 10.3390/ijms21145102 PMC7404208

