# Supplementary material for: Undifferentiated connective tissue disease: the diagnoses critically revised-experience of a single center
Source: Clin Exp Med. 2025 Mar 29;25(1):100. doi: 10.1007/s10238-025-01614-1 (PMC11954846; doi:10.1007/s10238-025-01614-1)
Supplement: Supplementary file 1 — Supplementary file1 (DOCX 24 KB) [file 10238_2025_1614_MOESM1_ESM.docx]

Supplemental tables

*Supplemental Table 1: demographic data of evolved and stable UCTD patients****.***

|  | Evolved n.33 | Stable n. 147 | p |
| --- | --- | --- | --- |
| F:M, (% F) | 31:2 (93.9) | 135:12 (91.8) | 1 |
| Caucasian (%) | 31 (93.9) | 135 (91.8) | 0.741 |
| Mean age at onset, years (SD) | 41.45 (13.4) | 43.54 (15.5) | 0.47 |
| Mean time at evolution or follow-up, years (SD) | 6.96 (4.45) | 8.51 (5) | 0.10 |

*Supplemental Table 2: laboratory data of evolved and stable UCTD patients****.***

|  | Evolved n.33 (%) | Stable n. 147 (%) | p |
| --- | --- | --- | --- |
| Hypergammaglobulinemia | 7 (21.21) | 14 (9.52) | 0.073 |
| Complement reduction | 4 (12.12) | 18 (12.24) | 1 |
| CRP elevation | 3 (9.09) | 13 (8.84) | 1 |
| Leukopenia | 3 (9.09) | 21 (14.28) | 0.576 |
| ESR elevation | 2 (6.06) | 10 (6.80) | 1 |
| Hemolytic anemia | 2 (6.06) | 13 (8.84) | 1 |
| Thrombocytopenia | 0 (0) | 13 (8.84) | 0.130 |

CRP: C-reactive protein; ESR: erythrocyte sedimentation rate

*Supplemental Table 3A: demographic data of RA-evolved and stable UCTD patients****.***

|  | RA-evolved n.3 | Stable n.147 | p |
| --- | --- | --- | --- |
| F/M, (% F) | 3/0 (100) | 135/12 (91.8) | 1 |
| Caucasian, (%) | 3 (100) | 135 (91.8) | 1 |
| Age at onset, mean, years (SD) | 52.67 (11.3) | 43.54 (15.5) | 0.31 |
| Mean follow-up medio or time to evolution, years (SD) | 6.81 (0.48) | 8.51 (5) | 0.56 |

*Supplemental Table 3B: laboratory data of RA-evolved and stable UCTD patients****.***

| ORIO | RA-evolved n.3 (%) | Stable n.147 (%) | p | OR (95IC) |
| --- | --- | --- | --- | --- |
| ANA | 3 (100) | 147 (100) | 1 |  |
| Isolated ANA | 0 (0) | 31 (21.09) | 1 |  |
| Multiple autoantibodies | 3 (100) | 116 (78.91) | 1 |  |
| Anti-ENA | 3 (100) | 81 (55.10) | 0.259 |  |
| Anti-Ro/SSA | 2 (75) | 56 (38.10) | 0.560 |  |
| Anti-U1RNP | 1 (33.33) | 13 (8.84) | 0.256 |  |
| ACPA | 3 (100) | 7/41 (17.07) | **0.009** | **Infinite** |
| RF | 3 (100) | 18/81 (22.22) | **0.014** | **Infinite** |

ANA: antinuclear antibodies; anti-ENA: anti-extractable nuclear antigen antibodies; ACPA: anti-cyclic citrullinated peptide antibodies; RF: rheumatoid factor. In bold statistically significant values with their 95% confidence interval.

*Supplemental Table 5: demographic and clinical data of pSS- evolved, SSC-evolved and SLE-evolved patients*

|  | pSS n.8 (%) | SLE n. 5 (%) | SSc n. 14 (%) | p |
| --- | --- | --- | --- | --- |
| Mean follow-up at evolution, years (SD) | 8.8 (3.4) | 7.2 (3) | 5.6 (5) | ns |
| Mean age at onset, years (SD) | 42.5 (10) | 30 (5) | 43 (16) | **0.05**  **(LES vs others)** |
| Xerostomia | 5 (62.5) | 0 | 3 (21.4) | ns |
| Xerophtalmia | 5 (62.5) | 0 | 5 (35.7) | ns |
| Arthralgias | 3 (37.5) | 1 (20) | 3 (21.4) | ns |
| Fatigue | 3 (37.5) | 2 (40) | 3 (21.4) | ns |
| Parotid swelling | 2 (25) | 0 | 0 | ns |
| RP | 2 (25) | 2 (40) | 14 (100) | **<0.0001**  **(SSc vs others)** |
| Autoimmune thyroidits | 2 (25) | 0 | 1 (7) | ns |
| VC changes | 2/2 (100) | nd | 11 (78.6) | ns |
| Arthritis | 1 (12.5) | 2 (40) | 2 (14.3) | ns |
| Photosensitivity | 1 (12.5) | 2 (40) | 2 (14.3) | ns |

RP: Raunaud’s phenomenon. In bold statistically significant values.

*Supplemental Table 6 : laboratory data of pSS- evolved, SSc-evolved and SLE-evolved patients*

|  | pSS n.8 (%) | SLE n. 5 (%) | SSc n. 14 (%) | p |
| --- | --- | --- | --- | --- |
| Hypergammaglobulinemia | 3 (37.50) | 2 (40) | 1 (7) | 0.07 |
| Complement reduction | 2 (25) | 2 (40) | 0 | ns |
| Leukopenia | 2 (25) | 1 (20) | 0 | ns |
| ESR elevation | 1 (12.50) | 0 | 1 (7) | ns |
| CRP elevation | 1 (12.50) | 1 (20) | 1 (7) | ns |
| ANA | 8 (100) | 5 (100) | 14 | ns |
| Isolated ANA | 0 (0) | 0 | 2 | ns |
| Multiple autoantibodies | 8 (100) | 5 (100) | 12 (85.7) | ns |
| Anti-ENA total | 7 (87.50) | 2 (40) * | 12 (85.7) | **0.05 (SLE vs others)** |
| Anti-Ro/SSA | 6 (75) | 1 (50) | 1 (8.3) * | **0.013 (SSc vs others)** |
| Anti-La/SSB | 1 (12.50) | 0 | 0 | ns |
| Anti-CENP-B | 1 (12.50) | 0 | 5 | ns |
| Sm | 0 | 1 | 0 | ns |
| Anti-dsDNA | 0 | 3 (60) | 0 | ns |
| aPL (at least 1+) | 2/6 (33.33) | 4 (80) | 3/12 (25) | ns |
| RF | 6/7 (85.71) | 0 | 0 | ns |

ESR: erythrocyte sedimentation rate; CRP: C-reactive protein; ANA: antinuclear antibodies; anti-ENA: anti-extractable nuclear antigen antibodies; Anti-dsDNA: anti-double strand DNA antibodies; aPL: antiphospholipid antibodies tests; RF: rheumatoid factor; In bold statistically significant values.
